# Supplementary material for: Achievement of Both Mechanical Properties and Intrinsic Self-Healing under Body Temperature in Polyurethane Elastomers: A Synthesis Strategy from Waterborne Polymers
Source: Polymers (Basel). 2020 Apr 24;12(4):989. doi: 10.3390/polym12040989 (PMC7240400; doi:10.3390/polym12040989)
Supplement: Supplementary file 1 [file polymers-12-00989-s001.pdf]

polymers

Supplementary Materials

Achievement of Both Mechanical Properties and Intrinsic Self-Healing under Body Temperature in Polyurethane Elastomers: A Synthesis Strategy from Waterborne Polymers

Liangdong Zhang [1], Teng Qiu [1,2], Xiting Sun [1], Longhai Guo [1,2,*], Lifan He [2,3], Jun Ye [2,3], Xiaoyu Li [1,2,3,*]

[1] State Key Laboratory of Organic-Inorganic Composites, Beijing University of Chemical Technology, Beijing 100029, PR China; zhangld_buct@163.com (L.Z.); qiuteng@mail.buct.edu.cn (T.Q.); xitingsun@163.com (X.S.)
[2] Beijing Engineering Research Center of Synthesis and Application of Waterborne Polymer, Beijing University of Chemical Technology, Beijing 100029, PR China; helf@mail.buct.edu.cn (L.H.); yejun@mail.buct.edu.cn (J.Y.)
[3] Key Laboratory of Carbon Fiber and Functional Polymers, Ministry of Education, Beijing University of Chemical Technology, Beijing 100029, PR China.
* Correspondence: guolh@mail.buct.edu.cn (L.G.); lixy@mail.buct.edu.cn (X.L.)

I. Literature Analysis of Self-healing PUR and WPUR

Table S1. Self-Healing PUR and Waterborne PUR.

Liter.	Polymer	Self-healing Moiety	Healing condition	σ_0 /MPa	H_σ /%	ε_0 /%	H_ε /%
[1]	PUR	Thiuram Disulfide	Visible Light 24 h	0.38	97	202	87
[2]	PUR	Diarylbibenzofuranone	RT 24h	0.08	100	500	100
[3]	PUR	Coumarin	UV (254 nm 1 min then 350 nm 90 min)	3.6	68	225	78
[4]	PUR	Aromatic disulfide	RT 24 h	0.81	97	3100	97
[5]	PUR	Diels−Alder bond	100 °C 1 h then 60 °C 7 days	33	100	264	100
[6]	PUR	hindered urea bond	37 °C 12 h	9.3	76	300	89
[7]	PUR	H-bond & π-π stacking	45 °C 2 h	1.1	100	60	95
[8]	PUR	Diarylbibenzofuranone	50 °C 12 h	0.8	100	880	100
[9]	PUR	Diselenide bond	Visible Light 48 h	1.3	70	375	35
[10]	PUR	aromatic Schiff	RT 2 h	0.18	98	260	96
[11]	PUR	Diels−Alder bond	60 °C 24 h	27	84	19	36
[12]	PUR	2,6-pyridinedicarboxamide coordinate to Fe^{3+}	RT 48 h	0.23	97	1850	95
[13]	PUR	2,2'-bipyridine-5,5'-dicarboxylic amide with Zn^{2+}	RT 48 h	0.65	76	310	76

[14]	PUR	Aliphatic disulfide	Sunlight 6 h	9.5	96	950	98
[15]	PUR	Aliphatic disulfide	80 °C	11	91	700	100
[16]	PUR	Urethane & Fe_3O_4	NIR (200 °C)	22.5	100	800	100
[17]	PUR	Aromatic disulfide	60 °C 24 h	7.7	98	590	95
[18]	PUR	Aliphatic disulfide	60 °C 6 h	5.1	100	730	100
[19]	PUR	H-bond	RT 48 h	1.7	76	1800	89
[20]	PUR	Aromatic disulfide	RT 2 h	6.8	88	923	100
[21]	PUR	Aliphatic disulfide	70 °C 12 h	25	87	1700	94
[22]	PUR	Aromatic disulfide	100 °C Microwave 10 min	32	74	1156	93
[23]	PUR	Aromatic disulfide	70 °C 20 min	1.1	78	1100	96
[24]	WPUR	Aromatic disulfide	80 °C 24 h	16.5	82	680	64
[25]	WPUR	Aliphatic disulfide	75 °C 20 min then 24 h RT	26	100	600	100
[26]	WPUR	Aliphatic disulfide	70 °C	27	90	900	NM
[27]	WPUR	Aliphatic disulfide & β-cyclodextrin	65 °C	19	90	NM	NM
[28]	WPUR	Diels−Alder bond	130 °C	13.1	86	742	82
[29]	WPUR	Aromatic disulfide	25 °C 48 h	4.5	85	128	89
[30]	WPUR	Aliphatic disulfide	65 °C	0.74	90.5	NM	NM
[31]	WPUR	Coumarin	UV 365 nm	1.25	160	419	78.7

σ_0: original ultimate tensile strength. ε_0: original elongation at break of polymer material. H_σ: healing efficiency of the ultimate tensile strength. H_ε: healing efficiency of elongation at break. NM: not mentioned, RT: room temperature.

II. NMR Analysis of P22-4 and P44-4

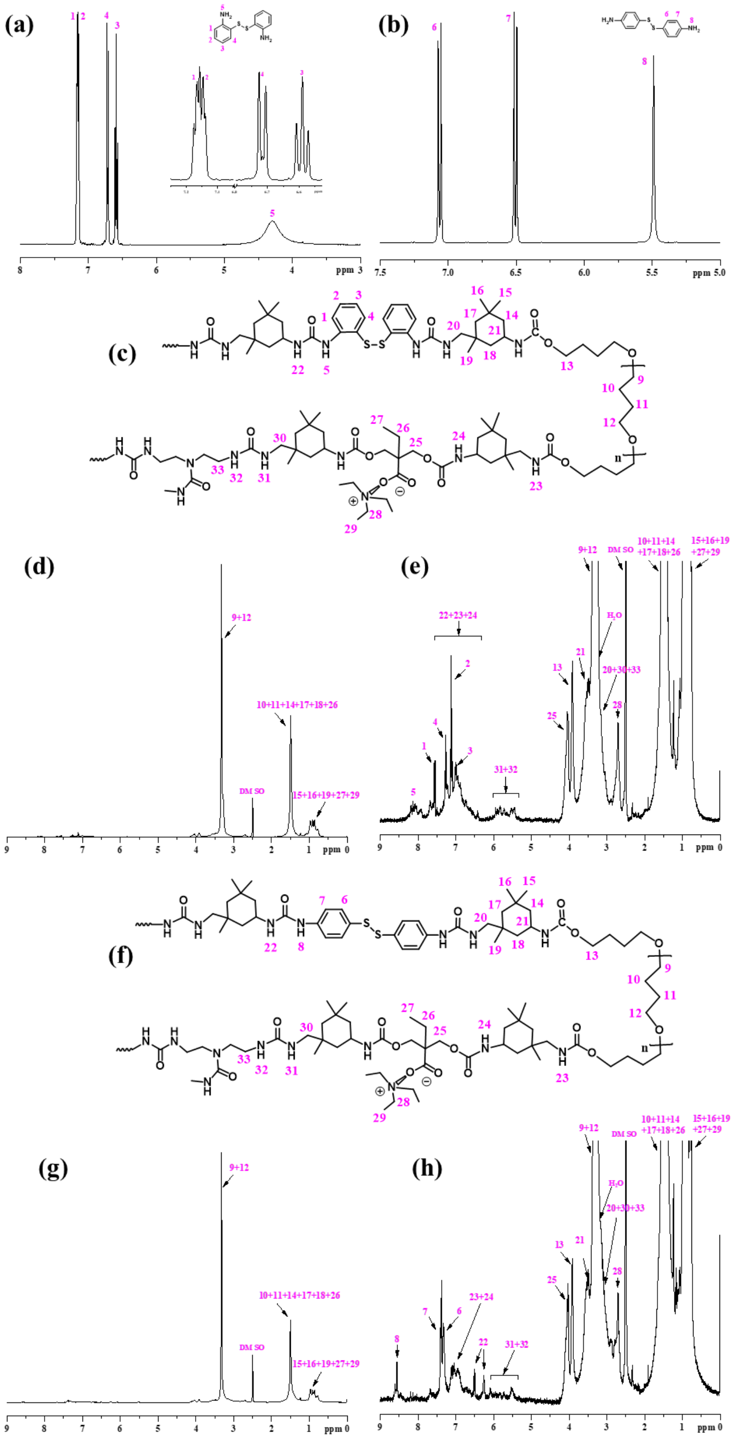

Figure S1. The ^{1}H NMR spectra and polymer chains models of the P22-4 and P44-4. (**a**) ^{1}H NMR spectrum of 22DTDA; (**b**) ^{1}H NMR spectrum of 44DTDA; (**c**) The polymer chain model of P22-4; (**d**) ^{1}H NMR spectrum of P22-4; (**e**) ^{1}H NMR spectrum of vertical amplification of P22-4; (**f**) The polymer chains model of P44-4; (**g**) ^{1}H-NMR spectrum of P44-4; (**h**) ^{1}H NMR spectrum of vertical amplification of P44-4.

To confirm the WPUR chain structure, the nuclear magnetic resonance (^{1}H NMR) was used and the testing results performed in Figure S1. In order to conveniently describe the corresponding peaks of different protons of structure in the polymer chain model, we use italics number from *1* to *33* to

refer to different protons in the chain structure. The ^{1}H NMR spectrum of 22DTDA was shown in Figure S1 (a), the aromatic protons of *1* and *2* performed resonance signals at 7.14–7.17 ppm, the aromatic protons of *3* and *4* exhibited triple and double splitting peaks at 6.59 ppm and 6.73 ppm, respectively, and the proton of *5* of -NH_2 groups shown a wide signal for H-bonding interaction. The ^{1}H-NMR spectrum of 44DTDA was exhibited in Figure S1 (b), the aromatic protons of *6* and *7* shown double splitting peaks at 7.05 ppm and 6.51 ppm, respectively, and proton of *5* of –NH_2 group played a peak at 5.48 ppm.

The ^{1}H NMR spectrum of P22-4 was exhibited in Figure S1 (d) and its vertical amplification was shown in Figure S1 (e). In Figure S1 (d), the strongest peak was the protons of the *9* and *12* from PTMEG at 3.29–3.35 ppm; the secondary strong peak was protons of –CH_2– groups from the *10*, *11*, *14*, *17*, *18* and *26* at 1.38-1.61 ppm; the resonance signal peaks at 0.74–1.05 ppm were attributed to the protons of the *15*, *16*, *19*, *27* and *29* of –CH_3 groups. In Figure S1 (e), the IPDI reacted with the PTMEG and DMBA to form the urethanes structures could be confirmed by the protons signals of –CH_2– flanked –NH–COO– group of the *13* and *22* at 3.92 ppm and 4.05 ppm, respectively; the peaks of aromatic protons of the *1*, *4*, *2*, and *3* were up to 7.55 ppm, 7.12 ppm, 7.01 ppm, and 7.27 ppm and –NH– proton of *5* were observed at 8.13 ppm, which were indicated that IPDI reacted with 22DTDA to form aromatic urea structure; the peak at 2.71 ppm corresponding the protons of *28* indicated formation of ammonium salt in polymer chains by neutralization of DMBA and TEA; the multi-peaks at 5.42–6.60 ppm were attributed to –NH– protons of aliphatic urea of the *31* and *32* formed by TETA reacted with IPDI; the –NH– protons of the *22*, *23* and *24* from the aromatic urea and urethane were performed wide and multi-peaks at 6.55-8.32 ppm due to H-bonding effect from urethane, urea, and DMSO solvent.

The ^{1}H NMR spectra of P44-4, in Figure S1 (g) and (h), were similar to the ^{1}H NMR spectra of P22-4, however, they performed a difference in low field from 5.2 to 8.7 ppm due to the different H bonds between the para and ortho structure. The aromatic protons of the *6* and *7* were a shift from the 7.05 ppm and 6.73 ppm at 44DTDA to the 7.32 ppm and 7.37 ppm at P44-4 with amine groups turn to urea group. The -NH- proton of *8* played a signal peak at 8.55 ppm. Moreover, the peaks of –NH– protons from the aliphatic urea and urethane in P44-4 were narrow and strength than that in P22-4, which indicates that the regularity and association of H-bonds in the WPUR material based on the para structure was higher than that based on ortho structure.

III. Supplement of GPC Analysis of P22 and P44 films

Table S2. Molecular mass of the WPUR dispersions.

Sample	$M_n \times 10^{-3}$ / g mol^{-1}	$M_w \times 10^{-3}$ / g mol^{-1}	M_w/M_n
P-0	82	162	1.96
P22-2	60	86	1.44
P44-2	67	123	1.73
P22-4	33	63	1.93
P44-4	43	75	1.73
P22-6	18	36	1.95
P22-8	12	21	1.77
P22-10	9.7	18	1.81

M_n: number average molecular mass. M_w: mass average molecular mass. M_w/M_n: molecular mass distribution.

The molecular mass of WPUR dispersions were measured using gel permeation chromatography (GPC). The test results were listed in Table S2. The molecular mass of DTDA was low than that of PTMEG. Thus, the number average molecular mass (M_n) of the synthesized WPUR decreases with the increasing addition of DTDA, which is consistent with the calculation results of the classical step polymerization theory (CSPT). Comparing the molecular mass of WPURs at the same content of the para and ortho structure, they had similar average number molecular mass and distributions.

IV. Supplement of Particle Size Distribution of P22-4 based on TEM

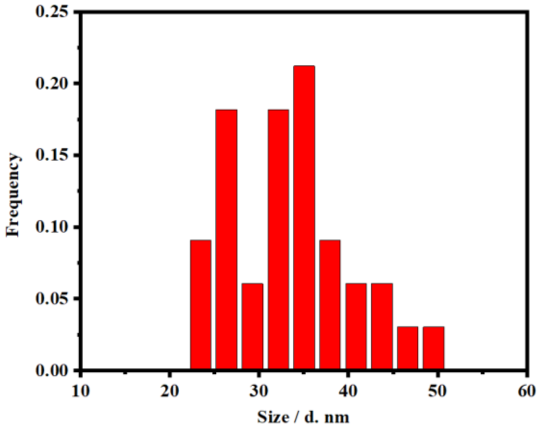

Figure S2. Particle size distribution of P22-4 based on TEM.

V. Supplement of Self-healing Performance of P22 and P44

The repeatable self-healing test was carried out as follow: (I) the tensile samples were completely cut by surgical knife; damaged samples were contacted carefully, slightly press to confirm adhesion, and transferred into 50 °C oven for 24 h; (III) the healed samples were cut in the position that (I) step damage and repeated (II) step of healing process; final the repeated healed samples were carried out tensile test to confirm healing ability and results in Figure S3 (a).

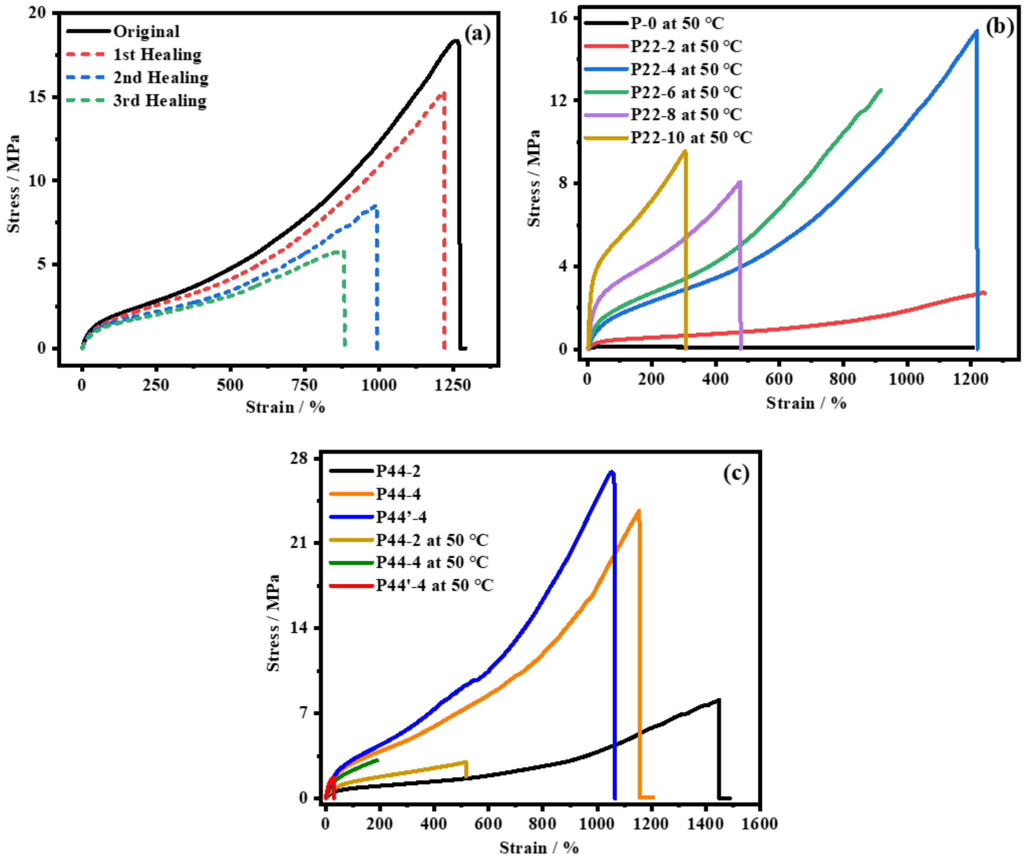

Figure S3. (**a**) typical tensile curves of P22-4 in repeated healing at 50 °C for 24 h; (**b**) typical tensile curves of P-0 and P22 films after healing at 50 °C for 24 h; (**c**) typical tensile curves of original P44-2, P44-4 and P44′-4 film and typical tensile curves of P44-2, P44-4 and P44′-4 film healing at 50 °C for 24 h.

VI. Supplement of FTIR Spectra of P22

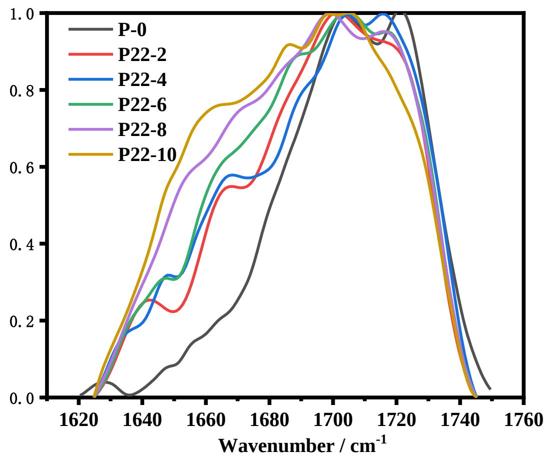

Figure S4. FTIR spectra of P-0 and P22 materials in the range from 1750 cm^{-1} to 1620 cm^{-1}.

VII. Supplement of Microphase Separation Section

Table S3. Glass transition temperature of soft, blend and hard phases of P22 and P44.

Sample	Soft phases / °C	Blend phases / °C	Hard phases / °C
P22-2	-43	no	123
P22-4	-42	11	114
P22-6	-44	30	129
P22-8	-44	30	138
P22-10	-44	42	140
P44-2	-39	96	167
P44-4	-50	70	180

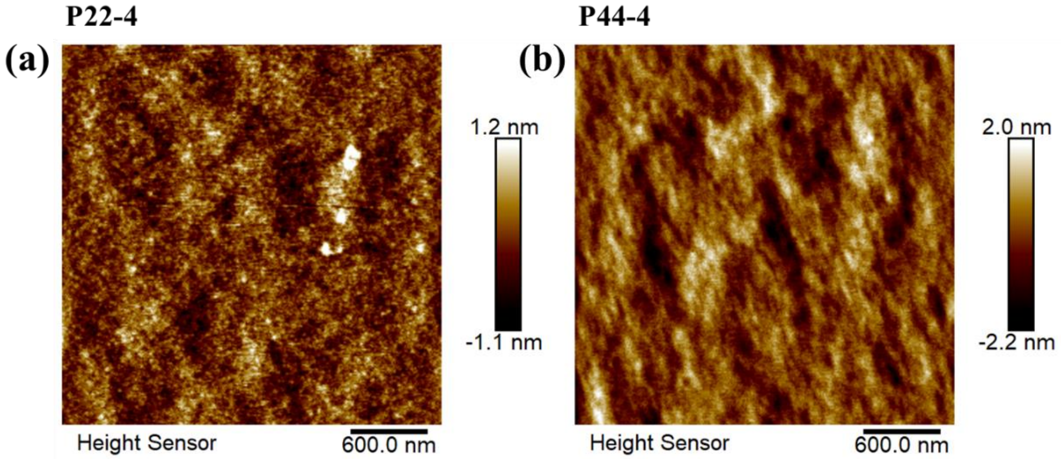

Figure S5. Atomic force microscopy height images (3 × 3 μm^2) of the surface of P22-4 (**a**) and P44-4 (**b**).

VIII. Thermostability Analysis

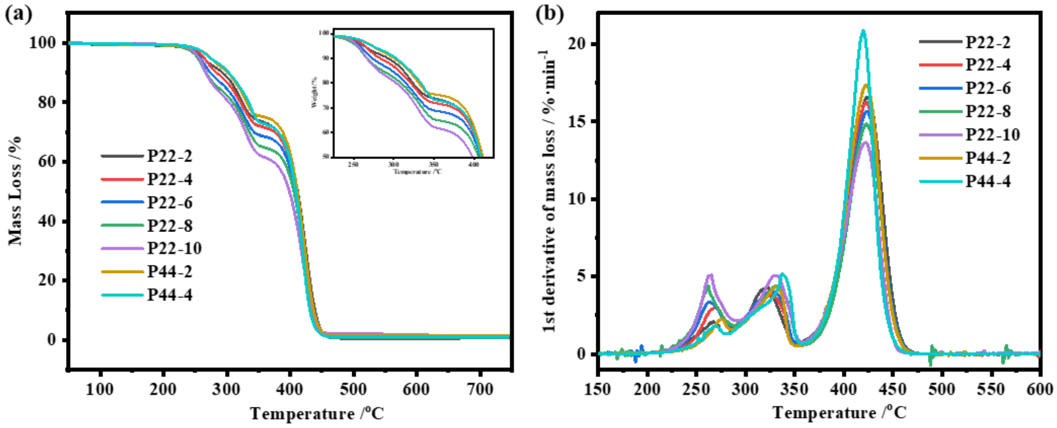

Figure S6. (**a**) Mass loss curves of the P22 and P44 films (**b**) The 1st derivative of mass loss of the P22 and P44 films.

The thermostability of the P22 and P44 films is evaluated by thermogravimetric analysis (TGA), corresponding mass loss curves in Figure S6. Compared the mass loss curves of P22 films in Figure S6 (a), thermostability decreased with the increase of 22DTDA content due to the low dissociation energy of aromatic disulfide. According to the 1st derivative of mass loss as a function of temperature in Figure S6 (b), the thermal degradation of the synthesized WPUR films was suffered three stages: the first stage about 220 °C to 285 °C, the second stage about 285 °C to 355 °C, and the third stage about 355 °C to 475 °C. In the first stage, the rate of mass loss increased with increasing 22DTDA content, and the ammonium salt from DMBA and TEA dissociates about 200 °C. Therefore, the first degradation stage may correspond to the degradation of structures including aromatic disulfide and TEA. In the second stage, the degree and rate of mass loss were closed in all samples with 22DTDA, which possibly indicated the degradation of IPDI-DMBA-IPDI segments. In the third stage, the mass loss was performed a grate decrease, which may reflect the degradation of IPDI-PTMEG-IPDI segments.

The mass loss rates of the P44 films were significantly slower than those of the P22 films, which suggested that para-UADS structure exhibits better thermostability than ortho-UADS structure.

References

1. Amamoto, Y.; Otsuka, H.; Takahara, A.; Matyjaszewski, K., Self-healing of covalently cross-linked polymers by reshuffling thiuram disulfide moieties in air under visible light. *Adv Mater* **2012,** *24* (29), 3975-80, 10.1002/adma.201201928.
2. Imato, K.; Nishihara, M.; Kanehara, T.; Amamoto, Y.; Takahara, A.; Otsuka, H., Self-healing of chemical gels cross-linked by diarylbibenzofuranone-based trigger-free dynamic covalent bonds at room temperature. *Angew Chem Int Ed Engl* **2012,** *51* (5), 1138-42, 10.1002/anie.201104069.
3. Ling, J.; Rong, M. Z.; Zhang, M. Q., Photo-stimulated self-healing polyurethane containing dihydroxyl coumarin derivatives. *Polymer* **2012,** *53* (13), 2691-2698, 10.1016/j.polymer.2012.04.016.
4. Rekondo, A.; Martin, R.; de Luzuriaga, A. R.; Cabanero, G.; Grande, H. J.; Odriozola, I., Catalyst-free room-temperature self-healing elastomers based on aromatic disulfide metathesis. *Mater Horiz* **2014,** *1* (2), 237-240, 10.1039/c3mh00061c.
5. Rivero, G.; Nguyen, L. T. T.; Hillewaere, X. K. D.; Du Prez, F. E., One-Pot Thermo-Remendable Shape Memory Polyurethanes. *Macromolecules* **2014,** *47* (6), 2010-2018, 10.1021/ma402471c.
6. Ying, H.; Zhang, Y.; Cheng, J., Dynamic urea bond for the design of reversible and self-healing polymers. *Nat Commun* **2014,** *5*, 3218, 10.1038/ncomms4218.
7. Feula, A.; Pethybridge, A.; Giannakopoulos, I.; Tang, X.; Chippindale, A.; Siviour, C. R.; Buckley, C. P.; Hamley, I. W.; Hayes, W., A Thermoreversible Supramolecular Polyurethane with Excellent Healing Ability at 45 °C. *Macromolecules* **2015,** *48* (17), 6132-6141, 10.1021/acs.macromol.5b01162.

8. Imato, K.; Takahara, A.; Otsuka, H., Self-Healing of a Cross-Linked Polymer with Dynamic Covalent Linkages at Mild Temperature and Evaluation at Macroscopic and Molecular Levels. *Macromolecules* **2015,** *48* (16), 5632-5639, 10.1021/acs.macromol.5b00809.
9. Ji, S.; Cao, W.; Yu, Y.; Xu, H., Visible-Light-Induced Self-Healing Diselenide-Containing Polyurethane Elastomer. *Adv Mater* **2015,** *27* (47), 7740-5, 10.1002/adma.201503661.
10. Lei, Z. Q.; Xie, P.; Rong, M. Z.; Zhang, M. Q., Catalyst-free dynamic exchange of aromatic Schiff base bonds and its application to self-healing and remolding of crosslinked polymers. *Journal of Materials Chemistry A* **2015,** *3* (39), 19662-19668, 10.1039/c5ta05788d.
11. Nguyen, L. T. T.; Truong, T. T.; Nguyen, H. T.; Le, L.; Nguyen, V. Q.; Van Le, T.; Luu, A. T., Healable shape memory (thio)urethane thermosets. *Polymer Chemistry* **2015,** *6* (16), 3143-3154, 10.1039/c5py00126a.
12. Li, C. H.; Wang, C.; Keplinger, C.; Zuo, J. L.; Jin, L.; Sun, Y.; Zheng, P.; Cao, Y.; Lissel, F.; Linder, C.; You, X. Z.; Bao, Z., A highly stretchable autonomous self-healing elastomer. *Nat Chem* **2016,** *8* (6), 618-24, 10.1038/nchem.2492.
13. Rao, Y. L.; Chortos, A.; Pfattner, R.; Lissel, F.; Chiu, Y. C.; Feig, V.; Xu, J.; Kurosawa, T.; Gu, X.; Wang, C.; He, M.; Chung, J. W.; Bao, Z., Stretchable Self-Healing Polymeric Dielectrics Cross-Linked Through Metal-Ligand Coordination. *J Am Chem Soc* **2016,** *138* (18), 6020-7, 10.1021/jacs.6b02428.
14. Xu, W. M.; Rong, M. Z.; Zhang, M. Q., Sunlight driven self-healing, reshaping and recycling of a robust, transparent and yellowing-resistant polymer. *Journal of Materials Chemistry A* **2016,** *4* (27), 10683-10690, 10.1039/c6ta02662a.
15. Xu, Y. R.; Chen, D. J., A Novel Self-Healing Polyurethane Based on Disulfide Bonds. *Macromolecular Chemistry and Physics* **2016,** *217* (10), 1191-1196, 10.1002/macp.201600011.
16. Yin, X. Y.; Zhang, Y.; Lin, P.; Liu, Y. P.; Wang, Z. K.; Yu, B.; Zhou, F.; Xue, Q. J., Highly efficient thermogenesis from Fe3O4 nanoparticles for thermoplastic material repair both in air and underwater. *Journal of Materials Chemistry A* **2017,** *5* (3), 1221-1232, 10.1039/c6ta09227f.
17. Yang, Y. L.; Lu, X.; Wang, W. W., A tough polyurethane elastomer with self-healing ability. *Materials & Design* **2017,** *127*, 30-36, 10.1016/j.matdes.2017.04.015.
18. Jian, X. X.; Hu, Y. W.; Zhou, W. L.; Xiao, L. Q., Self-healing polyurethane based on disulfide bond and hydrogen bond. *Polymers for Advanced Technologies* **2018,** *29* (1), 463-469, 10.1002/pat.4135.
19. Kang, J.; Son, D.; Wang, G. N.; Liu, Y.; Lopez, J.; Kim, Y.; Oh, J. Y.; Katsumata, T.; Mun, J.; Lee, Y.; Jin, L.; Tok, J. B.; Bao, Z., Tough and Water-Insensitive Self-Healing Elastomer for Robust Electronic Skin. *Adv Mater* **2018,** *30* (13), 1706846-1706854, 10.1002/adma.201706846.
20. Kim, S. M.; Jeon, H.; Shin, S. H.; Park, S. A.; Jegal, J.; Hwang, S. Y.; Oh, D. X.; Park, J., Superior Toughness and Fast Self-Healing at Room Temperature Engineered by Transparent Elastomers. *Adv Mater* **2018,** *30* (1), 1705145-1705153, 10.1002/adma.201705145.
21. Lai, Y.; Kuang, X.; Zhu, P.; Huang, M.; Dong, X.; Wang, D., Colorless, Transparent, Robust, and Fast Scratch-Self-Healing Elastomers via a Phase-Locked Dynamic Bonds Design. *Adv Mater* **2018,** *30* (38), e1802556, 10.1002/adma.201802556.
22. Ling, L.; Li, J. H.; Zhang, G. P.; Sun, R.; Wong, C. P., Self-Healing and Shape Memory Linear Polyurethane Based on Disulfide Linkages with Excellent Mechanical Property. *Macromolecular Research* **2018,** *26* (4), 365-373, 10.1007/s13233-018-6037-9.
23. Li, K.; Xu, Z. P.; Zhao, S. F.; Meng, X. Y.; Zhang, R. L.; Li, J. H.; Leng, J. F.; Zhang, G. P.; Cao, D. X.; Sun, R., Biomimetic, recyclable, highly stretchable and self-healing conductors enabled by dual reversible bonds. *Chemical Engineering Journal* **2019,** *371*, 203-212, 10.1016/j.cej.2019.04.053.
24. Nevejans, S.; Ballard, N.; Rivilla, I.; Fernandez, M.; Santamaria, A.; Reck, B.; Asua, J. M., Synthesis of mechanically strong waterborne poly(urethane-urea)s capable of self-healing at elevated temperatures. *European Polymer Journal* **2019,** *112*, 411-422, 10.1016/j.eurpolymj.2019.01.022.
25. Zhang, M.; Zhao, F.; Luo, Y., Self-Healing Mechanism of Microcracks on Waterborne Polyurethane with Tunable Disulfide Bond Contents. *ACS Omega* **2019,** *4* (1), 1703-1714, 10.1021/acsomega.8b02923.
26. Wan, T.; Chen, D. J., Mechanical enhancement of self-healing waterborne polyurethane by graphene oxide. *Progress in Organic Coatings* **2018,** *121*, 73-79, 10.1016/j.porgcoat.2018.04.016.
27. Wan, T.; Chen, D., Preparation of β-cyclodextrin reinforced waterborne polyurethane nanocomposites with excellent mechanical and self-healing property. *Composites Science and Technology* **2018,** *168*, 55-62, 10.1016/j.compscitech.2018.08.049.

28. Fang, Y. L.; Du, X. S.; Jiang, Y. X.; Du, Z. L.; Pan, P. T.; Cheng, X.; Wang, H. B., Thermal-Driven Self-Healing and Recyclable Waterborne Polyurethane Films Based on Reversible Covalent Interaction. *Acs Sustainable Chemistry & Engineering* **2018,** *6* (11), 14490-14500, 10.1021/acssuschemeng.8b03151.
29. Aguirresarobe, R. H.; Martin, L.; Fernandez-Berridi, M. J.; Irusta, L., Autonomic healable waterborne organic-inorganic polyurethane hybrids based on aromatic disulfide moieties. *Express Polymer Letters* **2017,** *11* (4), 266-277, 10.3144/expresspolymlett.2017.27.
30. Wan, T.; Chen, D., Synthesis and properties of self-healing waterborne polyurethanes containing disulfide bonds in the main chain. *Journal of Materials Science* **2016,** *52* (1), 197-207, 10.1007/s10853-016-0321-x.
31. Aguirresarobe, R. H.; Martin, L.; Aramburu, N.; Irusta, L.; Fernandez-Berridi, M. J., Coumarin based light responsive healable waterborne polyurethanes. *Progress in Organic Coatings* **2016,** *99*, 314-321, 10.1016/j.porgcoat.2016.06.011.
